# Validity and Reliability of POM-Checker for Measuring Shoulder Range of Motion in Healthy Participants: A Pilot Single-Center Comparative Study

**DOI:** 10.3390/mps6060114

**Published:** 2023-11-27

**Authors:** Hongmin Chu, Weonjin Kim, Seongsu Joo, Eunsik Park, Yeong Won Kim, Cheol-Hyun Kim, Sangkwan Lee

**Affiliations:** 1 Department of Internal Medicine and Neuroscience, College of Korean Medicine, Wonkwang University, Iksan 54538, Republic of Korea; hongminchu2@gmail.com; 2 Team Elysium Inc. R&D Center, Seoul 06682, Republic of Korea; wjkim@teamelysium.kr (W.K.); seongsu.joo@teamelysium.kr (S.J.); espark@teamelysium.kr (E.P.); ywon@teamelysium.kr (Y.W.K.); 3 Stroke Korean Medicine Research Center, Wonkwang University, Iksan 54538, Republic of Korea

**Keywords:** range of motion, pilot study, comparative study, shoulder joint

## Abstract

Background. The aim of this study was to compare shoulder movement measurements between a Kinect-based markerless ROM assessment device (POM-Checker) and a 3D motion capture analysis system (BTS SMART DX-400). Methods. This was a single-visit clinical trial designed to evaluate the validity and reliability of the POM-Checker. The primary outcome was to assess the equivalence between two measurement devices within the same set of participants, aiming to evaluate the validity of the POM-Checker compared to the gold standard device (3D Motion Analysis System). As this was a pilot study, six participants were included. Results. The intraclass correlation coefficient (ICC) and the corresponding 95% confidence intervals (CIs) were used to assess the reproducibility of the measurements. Among the 18 movements analyzed, 16 exhibited ICC values of >0.75, indicating excellent reproducibility. Conclusion. The results showed that the POM-checker is reliable and validated to measure the range of motion of the shoulder joint.

## 1. Introduction

The importance of objective measurements for assessing the health status of the musculoskeletal system has been increasingly recognized [1]. Manual muscle testing and joint range of motion (ROM) assessments are essential for eliminating subjective factors [2]. However, even with simple tools, such as goniometers or rulers for angle and length measurements, the potential for examiner bias and inconvenience remains, along with issues of reproducibility when using attachments on the body [3,4].

In particular, ROM assessment holds a critical position in the treatment of musculoskeletal disorders as it evaluates joint conditions and treatment effectiveness and forms the basis for disability assessments [5,6]. The POM-Checker (Team Elysium Inc., Seoul, Republic of Korea), which utilizes three-dimensional (3D) cameras and artificial intelligence, identifies joint positions, enabling the real-time tracking of joint movements and relative positions in 3D space [7]. The software uses a 3D sensor (RGB-D sensor) to identify the 3D location of body parts, enabling automatic joint angle measurements. Using the captured RGB-D images, a deep learning algorithm calculates the 3D joint coordinates [8]. Following this, angles between joint connections and reference vectors, such as the frontal axis, are measured. It measures the range of various joints, including the shoulder, hip, wrist, and cervical spine. As it utilizes a 3D sensor, it offers the advantages of high accuracy and reduced examination time by enabling measurements during the patient’s voluntary movements, eliminating the need for contact with the examiner. Furthermore, the device can measure the angles formed between the vectors representing both shoulders and the pelvis, knees, and the perpendicular vector of the floor, thus enabling postural assessment. Although conventional joint angle measuring devices, such as goniometers, visual estimation, and marker attachment systems, are prevalent, they suffer from high variability owing to manual operation [9]. In addition, motion and posture analysis systems require significant expertise and ample space, making their generalization challenging. Therefore, this device will offer convenient joint angle measurement for both examiners and patients.

However, to enhance the accuracy of the markerless Kinect-based system, the POM-Checker, developed by Team Elysium, incorporates a Kalman-filter-based algorithm alongside the joint recognition function of the Kinect SDK to track joint points. However, no clinical studies have assessed the accuracy of this device. Thus, our research team devised a protocol to compare the validity and reliability of the POM-Checker with marker attachment devices. A pilot study was conducted in accordance with the established protocol, and the findings are presented in detail in this report.

## 2. Materials and Methods

### 2.1. Measurement System

**Figure 1:**
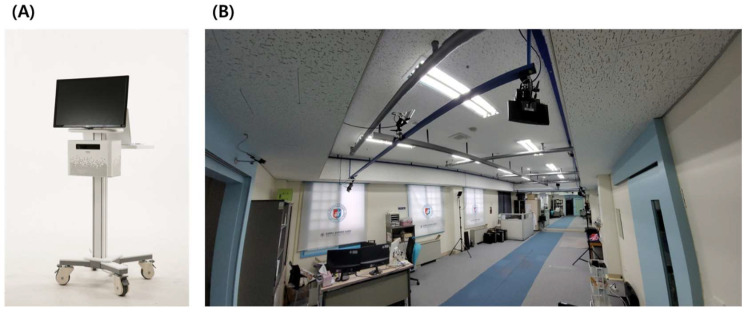
POM-Checker and motion capture system. The POM-Checker consists of a main computer, monitor, and Kinect. The 3D motion capture system has eight infrared cameras. (**A**) Anterior view of the POM-Checker. (**B**) Panoramic view of the BTS SMART DX-400 at GJWMC. This motion capture system has eight infrared cameras. Only seven cameras are only shown in the picture.

The POM-Checker obtained a product license (license number: 18-4334) for conformity assessment under the classification code A30130.01 (2), specifically for isokinetic testing and evaluation systems. This license was granted by the Korea Testing Certification, which holds the status of the National Official Professional Testing Research Institute (as depicted in Figure 1A).

For the 3D motion capture utilized in this study, the BTS SMART DX-400 system (BTS Bioengineering, Milano, Italy) was employed (Figure 1B). Operating at a rate of 100 fps, the BTS SMART DX-400 captured the motion data. In the global XYZ coordinates, the X-, Y-, and Z-axes were aligned with the patient’s anteroposterior direction, perpendicular to the ground, and right-left orientation, respectively. Calibration was performed due to the difference in measurement frame rates between the two devices. Firstly, we synchronized the computer times of both devices on the same local network and ensured that the measurement buttons were pressed simultaneously [10].

Regarding the shoulder, the determination of abduction or flexion involves observing changes in the Y values, which correspond to the vectors connecting the shoulder and elbow centers, as well as the vectors of the overall body trunk. Additionally, the X values, derived from the vectors connecting the shoulder center and the elbow center and the vectors of the entire body trunk, play a role in defining these movements. 

To measure shoulder joint movements, markers were attached to the shoulder area, according to the protocol. The marker positions are presented in the Protocol Paper [7]. 

### 2.2. Study Design

This pilot study was approved by the Institutional Review Board of Wonkwang University Korean Medicine Hospitals in Gwangju (GJWKH) (approval number: WKIRB2017-20, 23 January 2018). All the participants provided written informed consent after receiving an explanation of the objectives and characteristics of the study. This trial was registered with the Clinical Research Information Service (CRIS) of the Korea National Institute of Health (NIH), Republic of Korea (KCT0002851).

#### Study Procedure

The participants first entered the measurement room, where the 3D motion capture system and the POM-Checker were installed. To ensure proper joint recognition, the participants wore a sleeveless top provided by the research team. The participants stood at the center of the 3D motion capture room, and the POM-Checker was positioned at a distance of 3 m to encompass the entire body for measurement. 

Subsequently, Research Assistant 1 attached the markers to the participants and activated the POM-checker. Research Assistant 2 operated the 3D motion capture system simultaneously. The initiation of measurements on both the devices was calibrated to commence simultaneously when Research Assistant 2 initiated the 3D motion capture system [11,12]. After both devices were activated, the participant assumed a neutral position. Following a signal from Research Assistant 1, the target joint was slowly moved to reach a self-perceived angle of 90° before returning to a neutral position. 

The participants sequentially performed abduction and flexion movements of the right shoulder, followed by the same sequence for the left shoulder. After each movement was measured, participants were instructed to reset their poses. 

This pilot study was noninvasive and free from radiation exposure. This was a single-visit clinical trial designed to evaluate the validity of the medical devices. No specific hazards or adverse reactions were anticipated at the start of the study. In the event of any reported adverse reactions, the clinical trial was halted immediately and reported to the IRB of GJWKH. Measures were taken to ensure that discontinuation of the clinical trial would not impose any disadvantages or constraints on the participants. Even without adverse reactions, the participants had the option to discontinue their involvement in the study at any time.

### 2.3. Participants

The participants were recruited through posters or advertisements at Wonkwang University Korean Medicine Hospital in Gwangju (GJWKH). The participants were assigned a number of patients and were checked to determine whether they met all the criteria for enrollment.

#### 2.3.1. Inclusion Criteria

The participants were assigned unique identification numbers and assessed for eligibility based on specific enrollment criteria. The inclusion criteria specified individuals aged 18–39 years who willingly agreed to participate and provided written informed consent following a clear explanation of the clinical study’s objectives and characteristics. Additionally, eligible participants had no pre-existing musculoskeletal disorders of the shoulder joint.

#### 2.3.2. Exclusion Criteria

The exclusion criteria were as follows:

(1) Pregnancy;
(2) Individuals with conditions affecting the nervous, immune, respiratory, endocrine, or cardiovascular systems;
(3) Individuals diagnosed with tumors or mental illnesses;
(4) Those with existing musculoskeletal disorders or mechanical abnormalities, such as fractures or acute sprains in the shoulder joint;
(5) Individuals experiencing severe disability or significant pain around the shoulder joint.

### 2.4. Sample Size

The primary objective of this study was to assess the equivalence between two measurement devices (POM-Checker and 3D Motion Analysis System) within the same set of participants, aiming to evaluate the validity of the POM-Checker compared to the gold standard device (3D Motion Analysis System).

Drawing upon previous research that examined the validity of knee joint ROM assessments utilizing a goniometer, where a regression analysis between imaging data and an electro-goniometer exhibited a coefficient of determination (R^2^) of 0.99 and a standard deviation of 5°, we employed a paired *t*-test sample size calculation to determine an appropriate sample size. Setting the significance level (α) at 0.05, the statistical power (β) at 0.02, the effect size (using Cohen’s d; E) at 0.5, and utilizing S(Δ) = 0.71 (derived from S = 5.0 and r_within_ = 0.99), the calculation indicated a minimum sample size requirement of 15.83 participants. Considering the potential dropout rate of 4 participants, a total of 20 participants were selected for the clinical trial [6]. In this clinical trial, we targeted a total of 20 participants, with considerations made for potential dropout. The pilot study, designed as an initial step to assess the feasibility and validity of our research plan, content, and methodology, was conducted with 6 participants, representing 30% of the planned full trial size. This preliminary study aimed to provide insights and identify potential adjustments before proceeding with the main study.

### 2.5. Statistical Analysis

The Wilcoxon signed-rank test was conducted to assess differences in the POM-Checker test–retest consistency and to assess concordance between the values obtained from the BTS SMART DX-400 and POM-Checker. All analyses were performed using R version 1.0.143 software (R Studio, Boston, MA, USA). 

The intraclass correlation coefficient (ICC) and the corresponding 95% confidence intervals (CIs) were used to assess reproducibility of the measurements. The ICC was computed using the absolute agreement, two-way random effects model. ICC values less than 0.40, between 0.41 and 0.60, between 0.61 and 0.80, and greater than 0.81 are indicative of fair, moderate, substantial, and almost perfect reliability, respectively [13,14,15]. 

## 3. Results

### 3.1. Participants 

Of the included participants, three were men and three were women. The participants’ mean age, mean height, and mean weight were 26.7 ± 2.9 years, 162.2 ± 4.5 cm, and 60.2 ± 5.4 kg, respectively. No participants were excluded following interviews or the verification of comorbidities.

### 3.2. Correlation Analysis between POM-Checker and 3D Motion Capture Analysis (BTS SMART DX-400)

A correlation analysis was conducted by matching the measurements from the two devices. Unlike the analysis of static measurements in specific postures, this study aimed to present a correlation analysis of the values obtained during each participant’s continuous motion states. A correlation analysis was performed for right shoulder abduction, left shoulder abduction, right shoulder flexion, and left shoulder flexion, all of which demonstrated statistically significant correlations (Table 1).

Furthermore, to provide the average value of the correlation analysis, each correlation coefficient was transformed using Fisher’s Z. The mean of the Z values was calculated, and subsequently, back-transformation to the correlation coefficient was performed. After calculating the mean Z(r) values and back-transformation, the computed mean correlation coefficients were as follows: right abduction, 0.98; left abduction, 0.98; right flexion, 0.90; and left flexion, 0.90.

### 3.3. Mean Difference between POM-Checker and 3D Motion Capture Analysis (BTS SMART DX-400)

The mean difference and standard deviation of the measurements between the two devices for individual participants are presented in Table 2. Participants 1–5 exhibited mean differences within 3° for abduction, whereas for flexion, the mean difference exceeded 15° across all the participants. This discrepancy was attributed to noise during flexion measurements; the raw data are included in the Supplementary Materials. However, Participant 6 displayed considerable mean differences in both abduction and flexion movements. This was attributed to issues related to marker attachment and compensatory motion in this participant. Participant 6 was the tallest among the participants, making it challenging to accurately attach the markers at the skeletal landmarks. The substantial differences observed between the participants’ results may stem from these factors. 

### 3.4. Intraclass Correlation Coefficient (ICC)

The ICC values for both the POM-Checker and 3D motion capture analysis demonstrated high levels of agreement (Table 3). Values are presented as intraclass correlation coefficients (95% confidence interval). The ICC values were measured for a total of four movements among the six participants. Six movements were not included in the ICC calculation for the POM-Checker due to a 3-s delay in its initiation. Among the 18 movements analyzed using the motion capture analysis and POM-Checker, 16 exhibited ICC values of >0.75, indicating excellent reproducibility. One movement displayed an ICC value of >0.40, indicating fair-to-good reliability, whereas limited reproducibility was observed only in the right abduction of Participant 5. 

## 4. Discussion

The physical examination of the shoulder joint holds a paramount role in clinical assessment, offering insights into musculoskeletal function, identifying potential issues, and aiding in treatment planning [16]. Precise measurement of the range of motion (ROM) is a fundamental aspect of such examinations, providing crucial information about joint health and overall mobility [17,18,19]. Traditionally, goniometers and other angle-measuring tools have been employed for these assessments [20]. However, these conventional methods can be prone to inaccuracies due to various factors such as measurement errors, variability in anatomical landmarks, and discomfort experienced by patients during the examination process [21].

In this context, the emergence of markerless motion capture devices, such as Kinect-based systems, presents a convenient and promising solution [22]. These devices utilize advanced computer vision technology to track and analyze joint movements without the need for physical markers or attachments on the body [23,24]. This markerless approach not only simplifies the examination process for both clinicians and patients, but also addresses some of the limitations associated with traditional tools [25]. Its convenience lies in the non-invasive nature of the procedure, allowing for more natural movements and reducing patient discomfort. Furthermore, during our pilot study, we did not verify differences based on gender. However, as the number of participants will increase in the main clinical trial, we plan to assess whether there are accuracy variations based on gender as well.

Nevertheless, despite the benefits of markerless technology, there are still issues, particularly regarding the precision of angle readings [26,27]. The accuracy of joint angle estimates can be impacted by elements including occlusions, sensor noise, and algorithm restrictions. Furthermore, it might be challenging to precisely capture joint angles due to individual differences in body types and movement patterns [27]. Because of this, even if markerless devices have greater potential and offer increased convenience, extensive validation and improvements are required to guarantee their dependability and accuracy in clinical situations [26,28].

In view of these factors, we have devised a strategy to conduct a clinical trial for the Kinect-based device, the POM-Checker. This trial marks the inaugural clinical evaluation of the POM-Checker. This study aimed to compare shoulder movement measurements between a Kinect-based markerless ROM assessment device (POM-Checker) and a 3D motion capture analysis system (BTS SMART DX-400).

This pilot study is significant as it represents the first study to compare shoulder joint movements using the POM-Checker with a gold standard 3D motion capture system. Moreover, the strength of this study lies in conducting a comparative clinical trial using a device capable of tracking participant movements, rather than measuring the ROM in a static posture. However, this pilot clinical trial identified several aspects that require improvement in the trial design, execution process, and result analysis for the subsequent main trial.

We observed several issues related to the clinical trial protocol. In the POM-Checker user interface, a delay of approximately 3 s in angle measurement was observed after initiation of the measurement. Owing to the participants’ unfamiliarity with the clinical trial procedure, if they began their movement immediately upon hearing the measurement initiation signal, the angle measurement would start capturing the motion midway through the process of raising the arm to 90°. Furthermore, we conducted measurements after a single simulation to ensure better accuracy and to minimize examiner bias in the current clinical trial. However, in future trial designs, a combination of three measurements, followed by a single simulation with the most optimal motion values selected, should be adopted to mitigate these concerns.

Second, shoulder joint movement was measured. Standard movements for shoulder joint measurement can be affected by compensatory movements, such as lateral trunk bending. Although the examiners instructed the participants to execute precise movements, individuals familiar with compensatory motions may yield inaccurate measurements. This aspect requires further refinement in future studies.

Third, a correlation was observed in the test results between the POM-Checker and BTS SMART DX-400. Overall, a strong correlation was observed between the test results for the two devices. In the dynamic analysis, the mean difference in abduction movements was consistently <3° on both sides, except for one participant (Participant 6). The distinct differences were attributed to factors such as marker attachment and compensatory motion. Shoulder flexion movement measurements were challenged by the noise generated when the participants’ fingers approached the Kinect device. Adjusting the measurement setup to have participants face sideways, rather than in front, is expected to enhance accuracy in future evaluations.

Finally, although goniometers are widely and easily used for joint ROM measurements, their contact-based nature may lead to slight overestimation due to the examiner’s touch [29,30,31,32]. Therefore, future studies should include additional medical devices.

Studies of this nature encounter difficulties in comparing devices due to variations in the frame rates of devices employed for dynamic movement assessment. Subsequent trials should consider standardizing the measurement start and end times by first measuring static postures and then conducting dynamic measurements. Furthermore, statistical analyses, including Pearson’s correlation tests, were performed. However, future research should incorporate Bland–Altman plots for improved statistical evaluation.

## 5. Conclusions

This study represents an initial exploration of the reliability and validity of the POM-Checker, a Kinect-based device, for measuring the range of motion of the shoulder joint. Regarding the measurement of upper extremity angles, we conducted a comparison between the results obtained from a motion capture system and those from the 3D RGB-D system, the POM-Checker (Team Elysium Inc., Seoul, Republic of Korea). On average, our findings revealed the following correlation coefficients for shoulder joint movements: 0.98 for right abduction, 0.98 for left abduction, 0.90 for right flexion, and 0.90 for left flexion. Although the preliminary results indicate promise, it is important to note that this study has certain limitations due to its pilot nature. To establish a more robust understanding of the POM-Checker’s validity and reliability, future studies with an improved research design and a larger sample size are needed.

## Figures and Tables

**Table 1:** Correlation between the POM-Checker and BTS SMART DX-400.

	Rt Abduction	Lt Abduction	Rt Flexion	Lt Flexion
Participant 1 (F/26)	0.953 **	0.970 **	0.943 **	0.902 **
Participant 2 (F/25)	0.989 **	0.998 **	0.756 **	0.993 **
Participant 3 (M/25)	0.998 **	0.978 **	0.972 **	0.992 **
Participant 4 (F/33)	0.992 **	0.984 **	0.883 **	0.980 **
Participant 5 (M/26)	0.944 **	0.985 **	0.943 **	0.981 **
Participant 6 (M/25)	0.964 **	0.850 **	0.732 **	0.780 **

** *p* < 0.01.

**Table 2:** Mean difference (°) and standard deviation between POM-Checker and BTS SMART DX-400.

	Rt Abduction (°)	Lt Abduction (°)	Rt Flexion (°)	Lt Flexion (°)
Participant 1 (F/26)	0.201 (−2.438 to 2.840)	−0.125 (−3.826 to 3.575)	16.683 (14.647 to 18.720)	19.063 (14.270 to 23.856)
Participant 2 (F/25)	−1.148 (−1.756 to −0.540)	−0.984 (−1.656 to −0.312)	−20.969 (−23.812 to −18.127)	−15.722 (−17.887 to −13.558)
Participant 3 (M/25)	0.901 (0.450 to 1.352)	−2.406 (−2.918 to −1.894)	−18.363 (−19.559 to −17.167)	−18.961 (−19.769 to −18.126)
Participant 4 (F/33)	0.852 (0.449 to 1.255)	2.479 (2.028 to 2.931)	−16.573 (−17.302 to −15.845)	−16.191 (−17.169 to −15.213)
Participant 5 (M/26)	−1.430 (−2.151 to −0.709)	−2.709 (−3.083 to −2.336)	−20.977 (−21.618 to −20.336)	−19.603 (−20.202 to −19.005)
Participant 6 (M/25)	17.282 (16.587 to 17.978)	14.500 (13.237 to 15.764)	13.954 (11.979 to 15.930)	10.692 (8.838 to 12.546)

**Table 3:** Intraclass correlation coefficient (ICC) values of the results between POM-Checker and BTS SMART DX-400.

	Rt Abduction	Lt Abduction	Rt Flexion	Lt Flexion
Participant 1 (F/26)	N/A	0.980 (0.897 to 0.999)	N/A	0.971 (0.850 to 0.999)
Participant 2 (F/25)	N/A	N/A	0.986 (0.928 to 0.999)	0.930 (0.639 to 0.999)
Participant 3 (M/25)	N/A	0.580 (−1.138 to 0.999)	0.988 (0.943 to 0.999)	0.993 (0.963 to 0.999)
Participant 4 (F/33)	N/A	0.763 (−0.205 to 0.999)	0.996 (0.979 to 0.999)	0.989 (0.945 to 0.999)
Participant 5 (M/26)	0.076 (−3.699 to 0.999)	0.690 (−0.574 to 0.999)	0.997 (0.984 to 0.999)	0.996 (0.978 to 0.999)
Participant 6 (M/25)	0.990 (0.945 to 0.999)	0.988 (0.939 to 0.999)	0.987 (0.932 to 0.999)	0.977 (0.880 to 0.999)

## Data Availability

The datasets used and/or analyzed during the current study are available from the corresponding author on reasonable request.

## Supplementary Materials

The following supporting information can be downloaded at: https://www.mdpi.com/article/10.3390/mps6060114/s1.
