# Supplementary material for: Validity and Reliability of POM-Checker for Measuring Shoulder Range of Motion in Healthy Participants: A Pilot Single-Center Comparative Study
Source: Methods Protoc. 2023 Nov 27;6(6):114. doi: 10.3390/mps6060114 (PMC10745328; doi:10.3390/mps6060114)
Supplement: Supplementary file 1 [file mps-06-00114-s001.zip › mps-2605079-supplementary.pptx]

## Slide 1
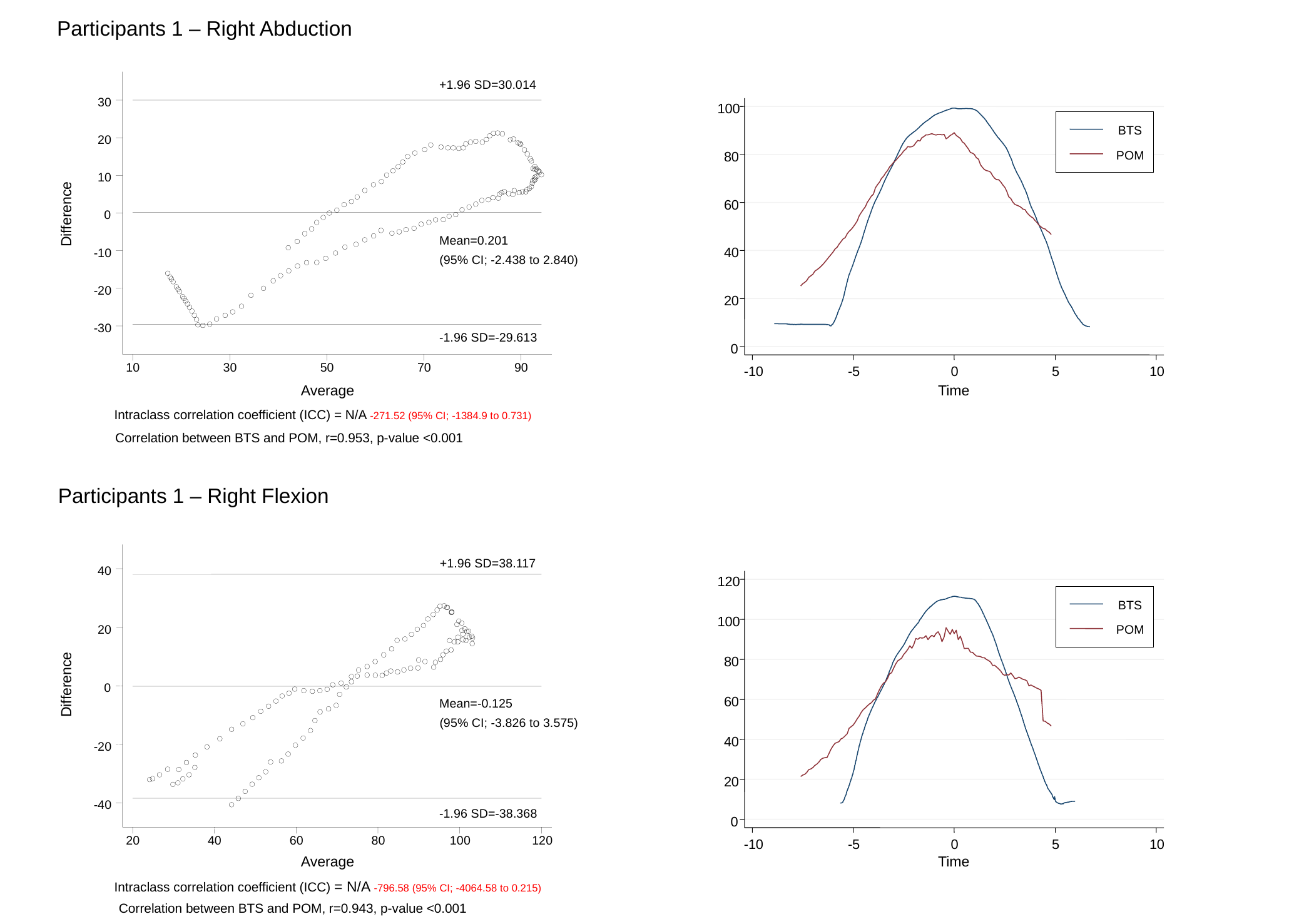

Participants 1 – Right Abduction
+1.96 SD=30.014
30
100
BTS
POM
20
80
10
60
Difference
0
Mean=0.201
40
-10
(95% CI; -2.438 to 2.840)
-20
20
-30
-1.96 SD=-29.613
0
10
30
50
70
90
-10
-5
0
5
10
Average
Time
Intraclass correlation coefficient (ICC) = N/A -271.52 (95% CI; -1384.9 to 0.731)
Correlation between BTS and POM, r=0.953, p-value <0.001
Participants 1 – Right Flexion
+1.96 SD=38.117
40
120
BTS
POM
100
20
80
Difference
0
60
Mean=-0.125
(95% CI; -3.826 to 3.575)
40
-20
20
-40
-1.96 SD=-38.368
0
20
40
60
80
100
120
-10
-5
0
5
10
Average
Time
Intraclass correlation coefficient (ICC) = N/A -796.58 (95% CI; -4064.58 to 0.215)
Correlation between BTS and POM, r=0.943, p-value <0.001

## Slide 2
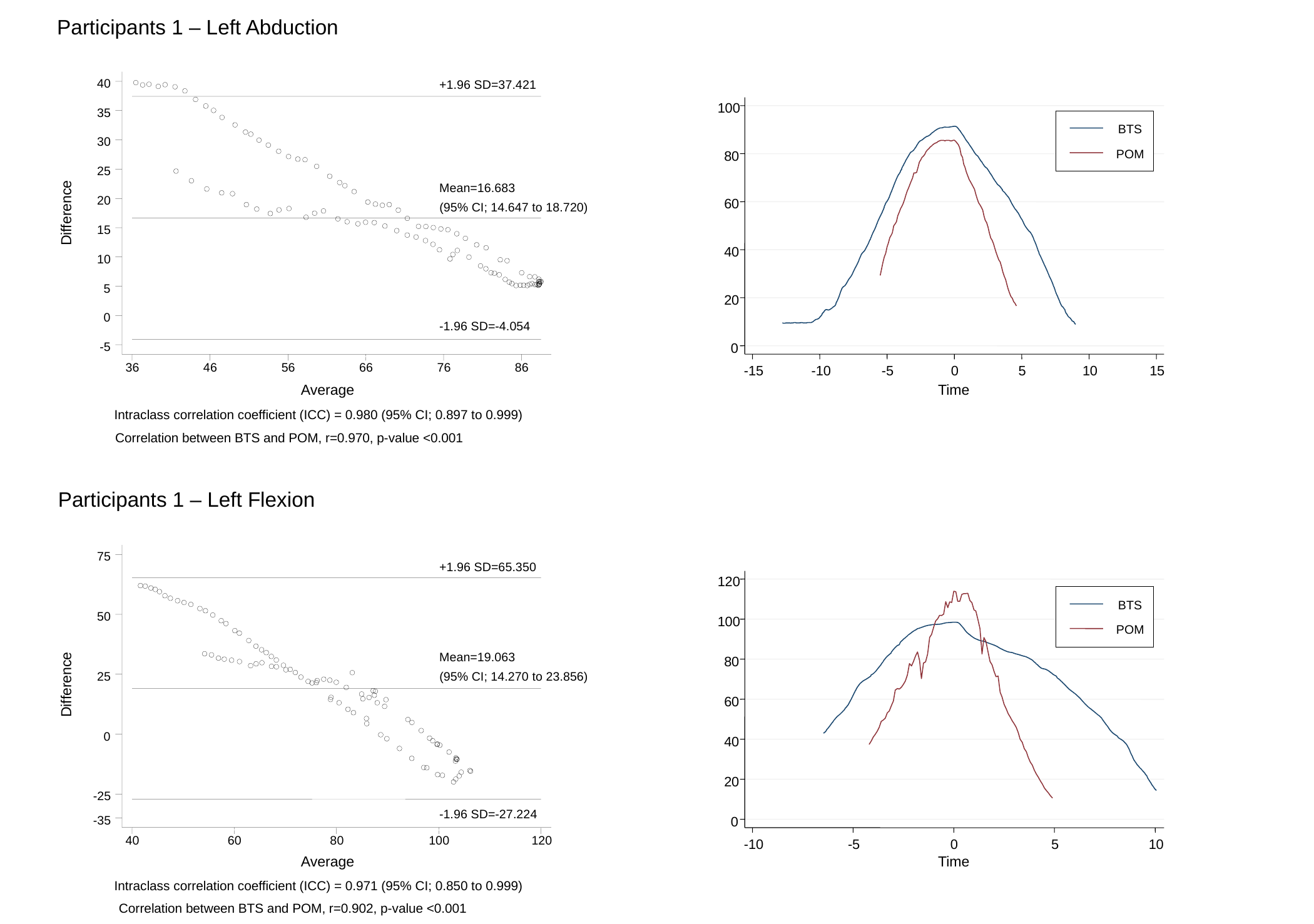

Participants 1 – Left Abduction
40
+1.96 SD=37.421
Mean=16.683
(95% CI; 14.647 to 18.720)
-1.96 SD=-4.054
Intraclass correlation coefficient (ICC) = 0.980 (95% CI; 0.897 to 0.999)
Correlation between BTS and POM, r=0.970, p-value <0.001
+1.96 SD=65.350
Mean=19.063
(95% CI; 14.270 to 23.856)
-1.96 SD=-27.224
Intraclass correlation coefficient (ICC) = 0.971 (95% CI; 0.850 to 0.999)
Correlation between BTS and POM, r=0.902, p-value <0.001
100
35
BTS
POM
30
80
25
20
60
Difference
15
40
10
5
20
0
-5
0
36
46
56
66
76
86
-15
-10
-5
0
5
10
15
Average
Time
Participants 1 – Left Flexion
75
120
BTS
POM
50
100
80
25
Difference
60
0
40
20
-25
-35
0
40
60
80
100
120
-10
-5
0
5
10
Average
Time

## Slide 3
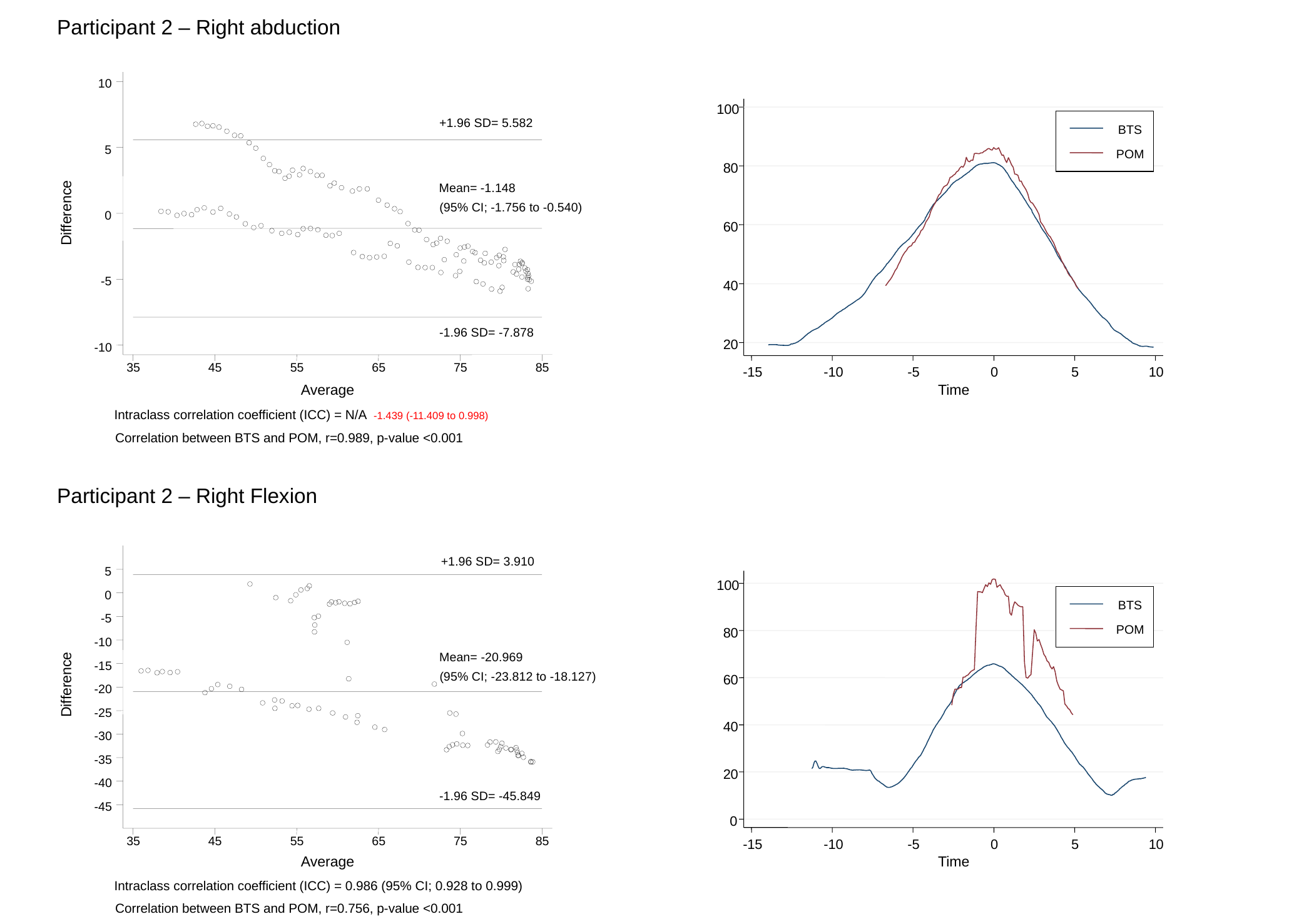

Participant 2 – Right abduction
10
100
+1.96 SD= 5.582
BTS
5
POM
80
Mean= -1.148
(95% CI; -1.756 to -0.540)
Difference
0
60
-5
40
-1.96 SD= -7.878
20
-10
35
45
55
65
75
85
-15
-10
-5
0
5
10
Average
Time
Intraclass correlation coefficient (ICC) = N/A -1.439 (-11.409 to 0.998)
Correlation between BTS and POM, r=0.989, p-value <0.001
Participant 2 – Right Flexion
+1.96 SD= 3.910
5
100
BTS
POM
0
-5
80
-10
Mean= -20.969
-15
(95% CI; -23.812 to -18.127)
60
Difference
-20
-25
40
-30
-35
20
-40
-1.96 SD= -45.849
-45
0
35
45
55
65
75
85
-15
-10
-5
0
5
10
Average
Time
Intraclass correlation coefficient (ICC) = 0.986 (95% CI; 0.928 to 0.999)
Correlation between BTS and POM, r=0.756, p-value <0.001

## Slide 4
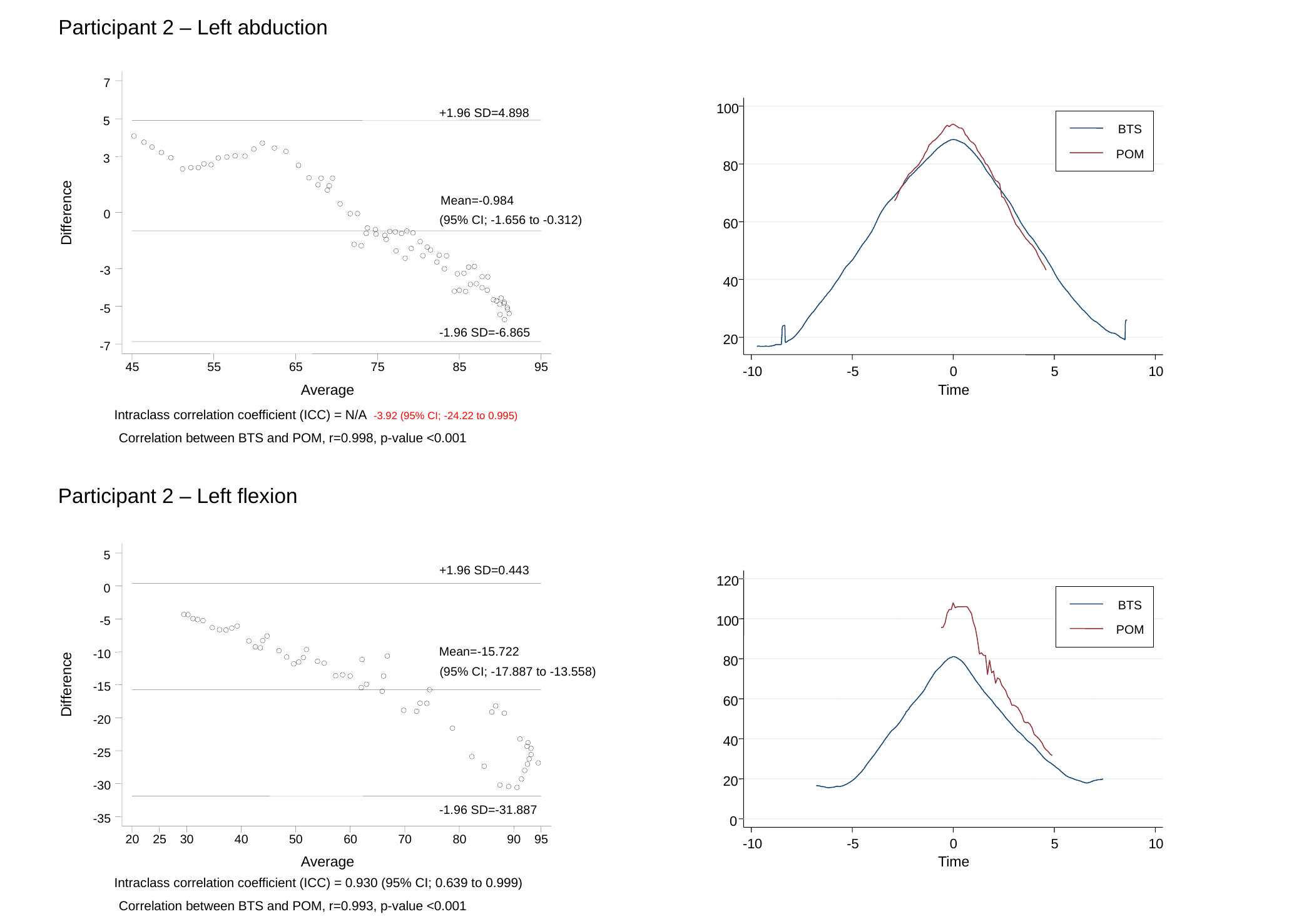

Participant 2 – Left abduction
7
100
+1.96 SD=4.898
BTS
POM
 5
 3
80
Mean=-0.984
Difference
0
(95% CI; -1.656 to -0.312)
60
-3
-5
40
-1.96 SD=-6.865
20
-7
45
55
65
75
85
95
-10
-5
0
5
10
Average
Time
Intraclass correlation coefficient (ICC) = N/A -3.92 (95% CI; -24.22 to 0.995)
Correlation between BTS and POM, r=0.998, p-value <0.001
Participant 2 – Left flexion
5
+1.96 SD=0.443
120
0
BTS
POM
100
-5
Mean=-15.722
-10
80
(95% CI; -17.887 to -13.558)
Difference
-15
60
-20
40
-25
20
-30
-1.96 SD=-31.887
-35
0
20
25
30
40
50
60
70
80
90
95
-10
-5
0
5
10
Average
Time
Intraclass correlation coefficient (ICC) = 0.930 (95% CI; 0.639 to 0.999)
Correlation between BTS and POM, r=0.993, p-value <0.001

## Slide 5
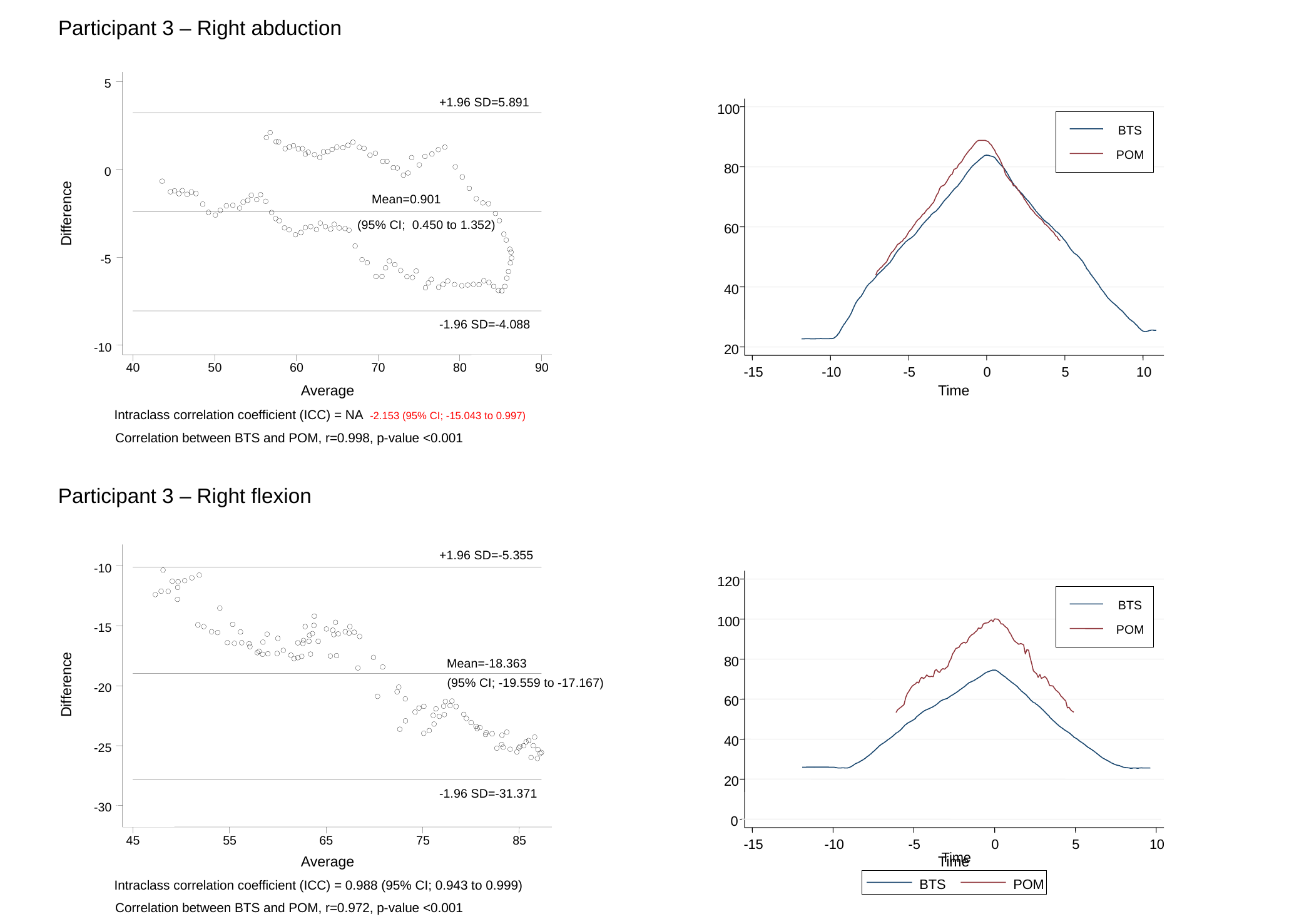

Participant 3 – Right abduction
5
+1.96 SD=5.891
100
BTS
POM
80
0
Mean=0.901
Difference
(95% CI; 0.450 to 1.352)
60
-5
40
-1.96 SD=-4.088
-10
20
40
50
60
70
80
90
-15
-10
-5
0
5
10
Average
Time
Intraclass correlation coefficient (ICC) = NA -2.153 (95% CI; -15.043 to 0.997)
Correlation between BTS and POM, r=0.998, p-value <0.001
Participant 3 – Right flexion
+1.96 SD=-5.355
-10
120
BTS
POM
100
-15
80
Mean=-18.363
(95% CI; -19.559 to -17.167)
Difference
-20
60
40
-25
20
-1.96 SD=-31.371
-30
0
45
55
65
75
85
-15
-10
-5
0
5
10
Time
Average
Time
Intraclass correlation coefficient (ICC) = 0.988 (95% CI; 0.943 to 0.999)
BTS
POM
Correlation between BTS and POM, r=0.972, p-value <0.001

## Slide 6
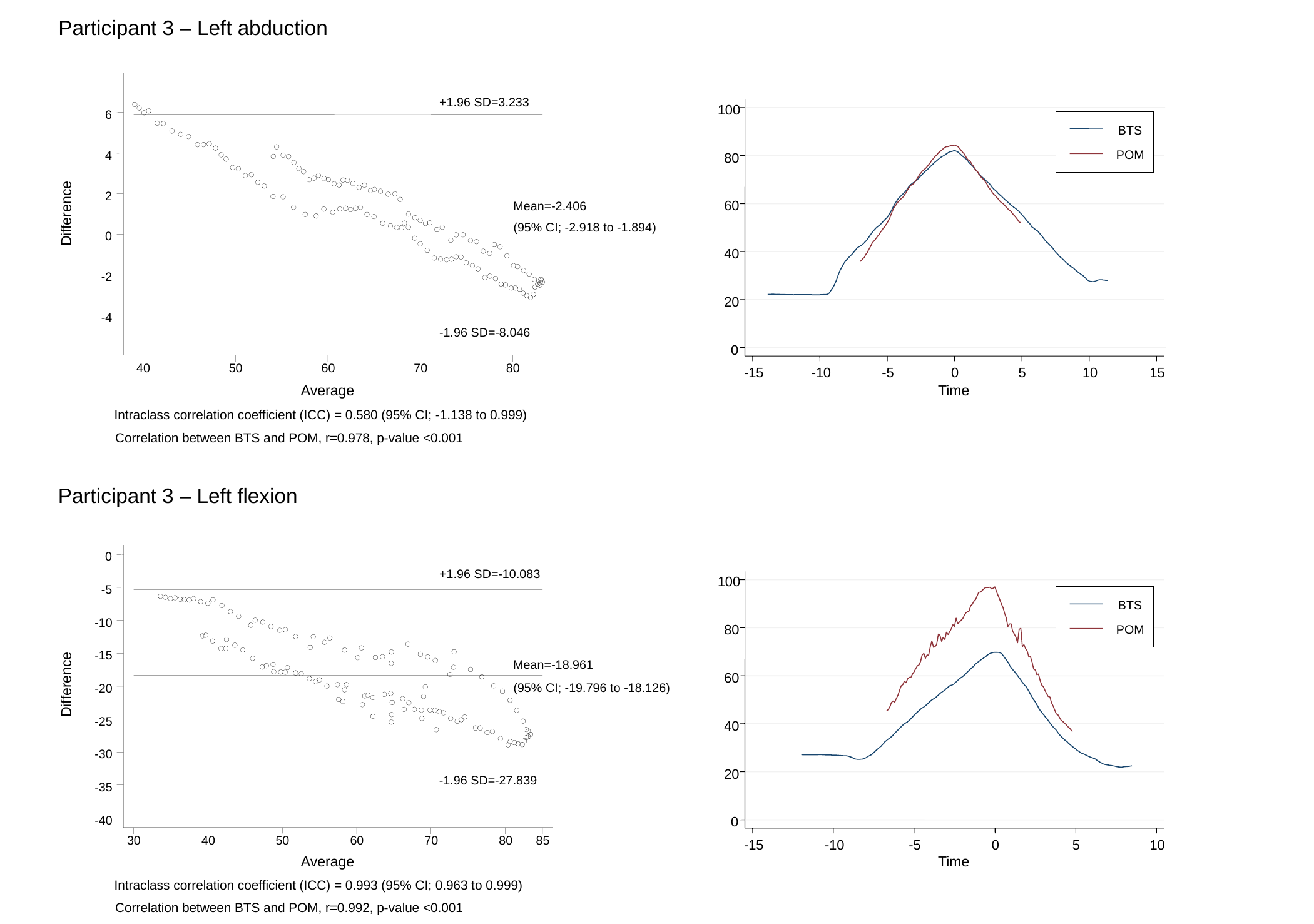

Participant 3 – Left abduction
+1.96 SD=3.233
100
6
BTS
POM
4
80
2
60
Mean=-2.406
Difference
(95% CI; -2.918 to -1.894)
0
40
-2
20
-4
-1.96 SD=-8.046
0
40
50
60
70
80
-15
-10
-5
0
5
10
15
Average
Time
Intraclass correlation coefficient (ICC) = 0.580 (95% CI; -1.138 to 0.999)
Correlation between BTS and POM, r=0.978, p-value <0.001
Participant 3 – Left flexion
0
+1.96 SD=-10.083
100
-5
BTS
POM
-10
80
-15
Mean=-18.961
60
Difference
(95% CI; -19.796 to -18.126)
-20
-25
40
-30
-35
20
-1.96 SD=-27.839
-40
0
30
40
50
60
70
80
85
-15
-10
-5
0
5
10
Average
Time
Intraclass correlation coefficient (ICC) = 0.993 (95% CI; 0.963 to 0.999)
Correlation between BTS and POM, r=0.992, p-value <0.001

## Slide 7
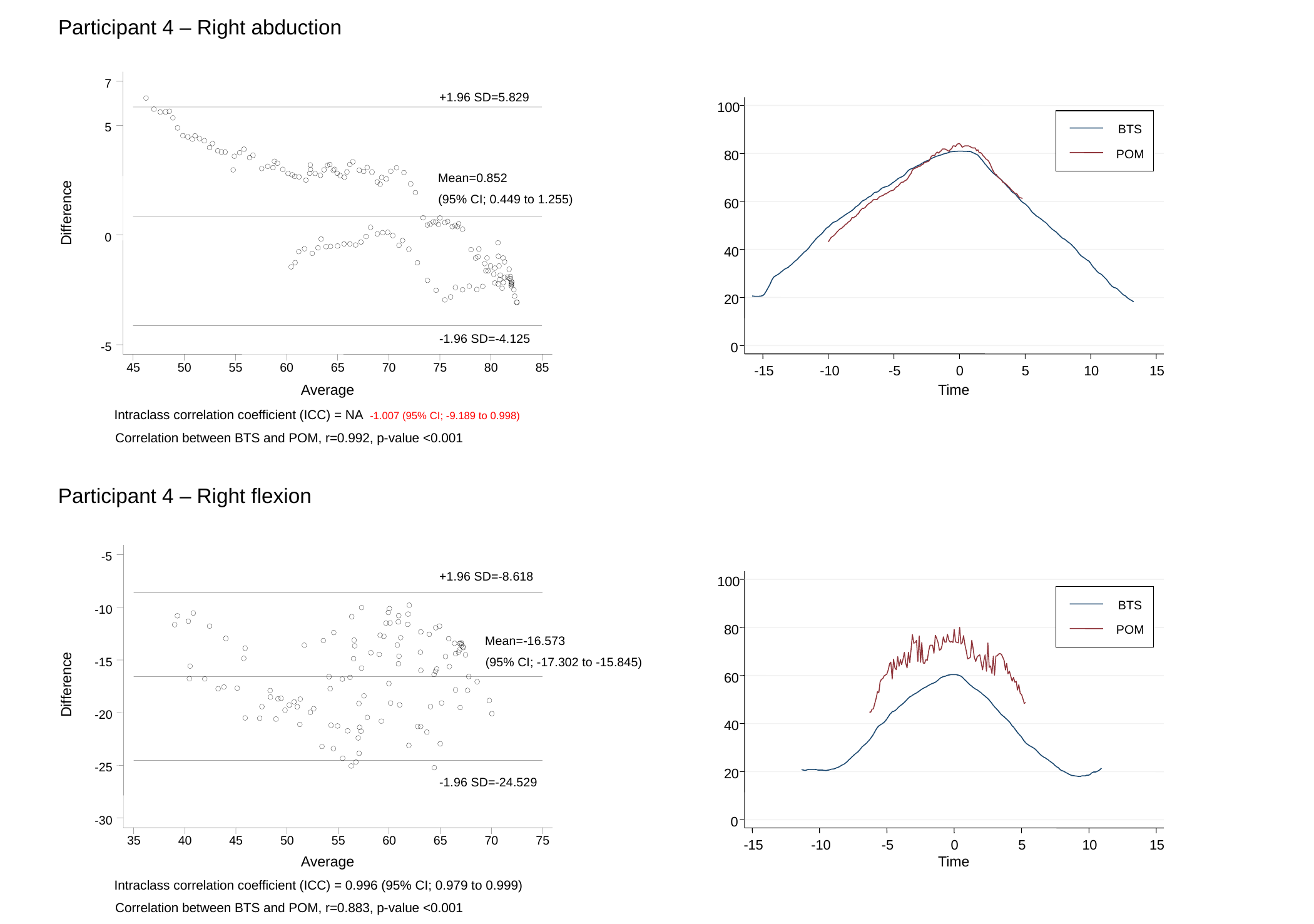

Participant 4 – Right abduction
7
+1.96 SD=5.829
100
BTS
POM
5
80
Mean=0.852
Difference
Average
Time
(95% CI; 0.449 to 1.255)
60
0
40
20
-1.96 SD=-4.125
-5
0
45
50
55
60
65
70
75
80
85
-15
-10
-5
0
5
10
15
Intraclass correlation coefficient (ICC) = NA -1.007 (95% CI; -9.189 to 0.998)
Correlation between BTS and POM, r=0.992, p-value <0.001
Participant 4 – Right flexion
-5
+1.96 SD=-8.618
100
BTS
POM
-10
80
Mean=-16.573
Difference
Average
Time
-15
(95% CI; -17.302 to -15.845)
60
-20
40
-25
20
-1.96 SD=-24.529
-30
0
35
40
45
50
55
60
65
70
75
-15
-10
-5
0
5
10
15
Intraclass correlation coefficient (ICC) = 0.996 (95% CI; 0.979 to 0.999)
Correlation between BTS and POM, r=0.883, p-value <0.001

## Slide 8
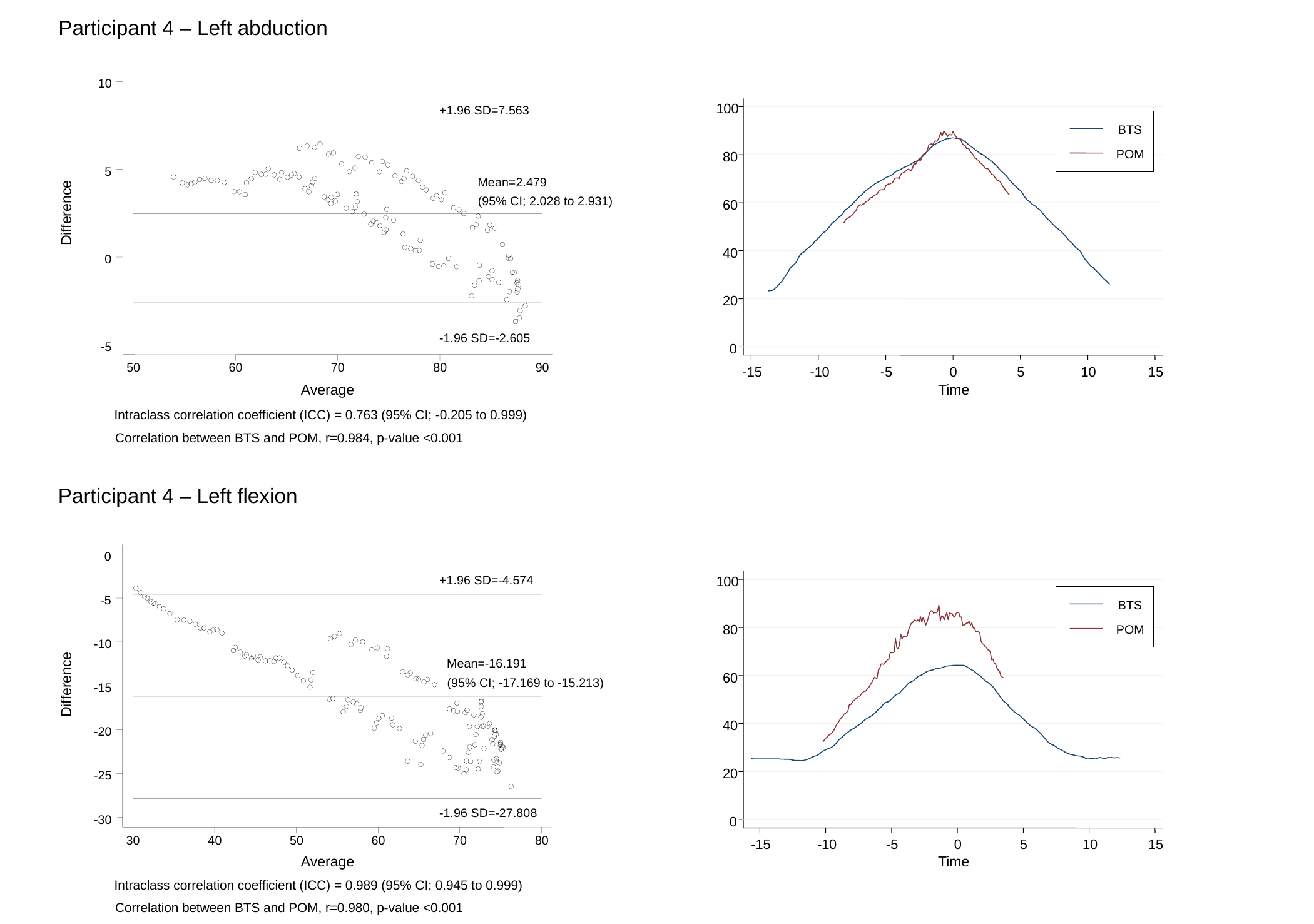

Participant 4 – Left abduction
10
100
+1.96 SD=7.563
BTS
POM
80
5
Mean=2.479
Difference
Average
Time
(95% CI; 2.028 to 2.931)
60
40
0
20
-1.96 SD=-2.605
-5
0
50
60
70
80
90
-15
-10
-5
0
5
10
15
Intraclass correlation coefficient (ICC) = 0.763 (95% CI; -0.205 to 0.999)
Correlation between BTS and POM, r=0.984, p-value <0.001
Participant 4 – Left flexion
0
+1.96 SD=-4.574
100
BTS
POM
-5
80
-10
Difference
Average
Time
Mean=-16.191
60
(95% CI; -17.169 to -15.213)
-15
40
-20
20
-25
-1.96 SD=-27.808
-30
0
30
40
50
60
70
80
-15
-10
-5
0
5
10
15
Intraclass correlation coefficient (ICC) = 0.989 (95% CI; 0.945 to 0.999)
Correlation between BTS and POM, r=0.980, p-value <0.001

## Slide 9
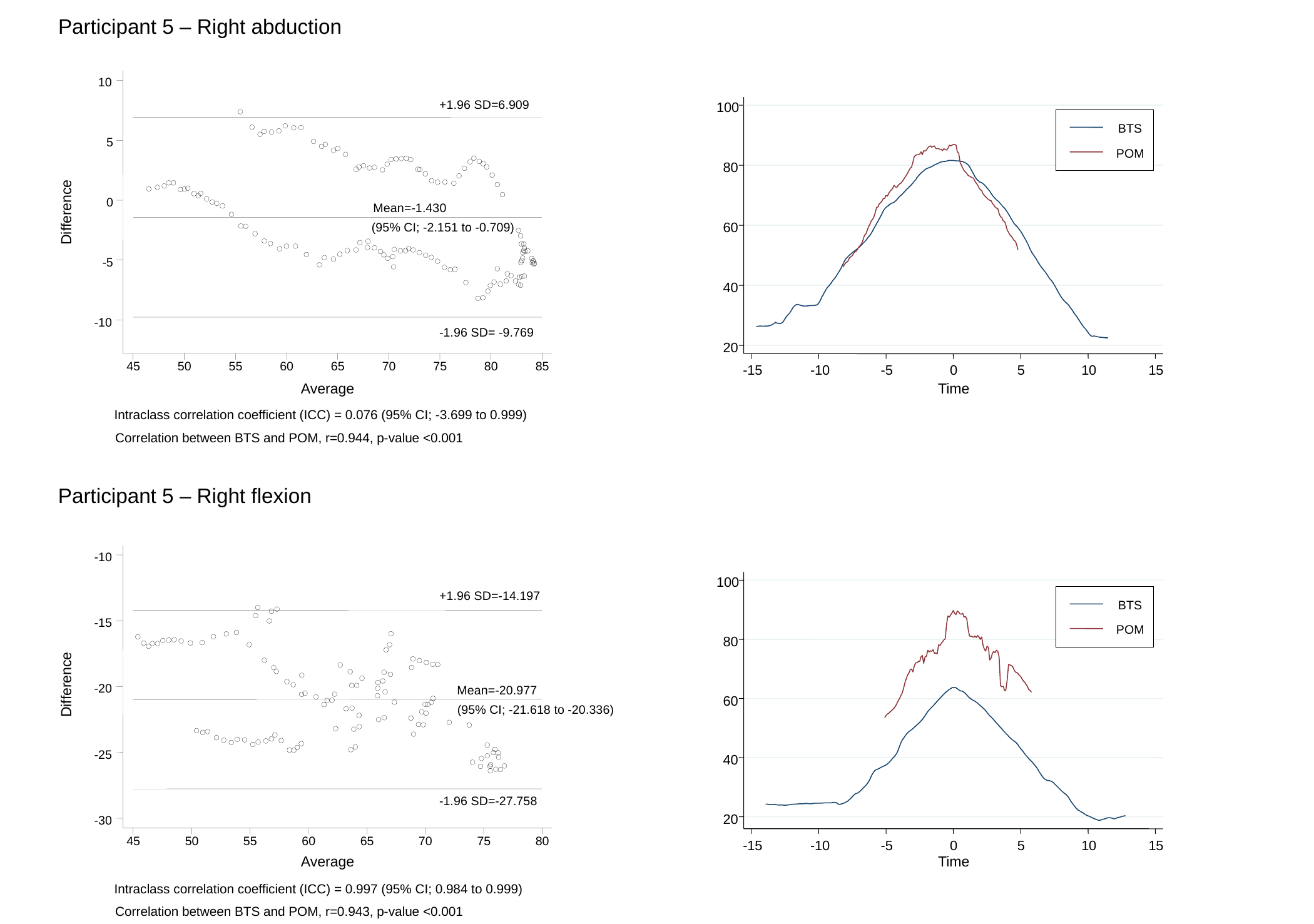

Participant 5 – Right abduction
10
+1.96 SD=6.909
100
BTS
POM
5
80
Difference
Average
Time
0
Mean=-1.430
60
(95% CI; -2.151 to -0.709)
-5
40
-10
-1.96 SD= -9.769
20
45
50
55
60
65
70
75
80
85
-15
-10
-5
0
5
10
15
Intraclass correlation coefficient (ICC) = 0.076 (95% CI; -3.699 to 0.999)
Correlation between BTS and POM, r=0.944, p-value <0.001
Participant 5 – Right flexion
-10
100
BTS
POM
+1.96 SD=-14.197
-15
80
Difference
Average
Time
-20
Mean=-20.977
60
(95% CI; -21.618 to -20.336)
-25
40
-1.96 SD=-27.758
20
-30
45
50
55
60
65
70
75
80
-15
-10
-5
0
5
10
15
Intraclass correlation coefficient (ICC) = 0.997 (95% CI; 0.984 to 0.999)
Correlation between BTS and POM, r=0.943, p-value <0.001

## Slide 10
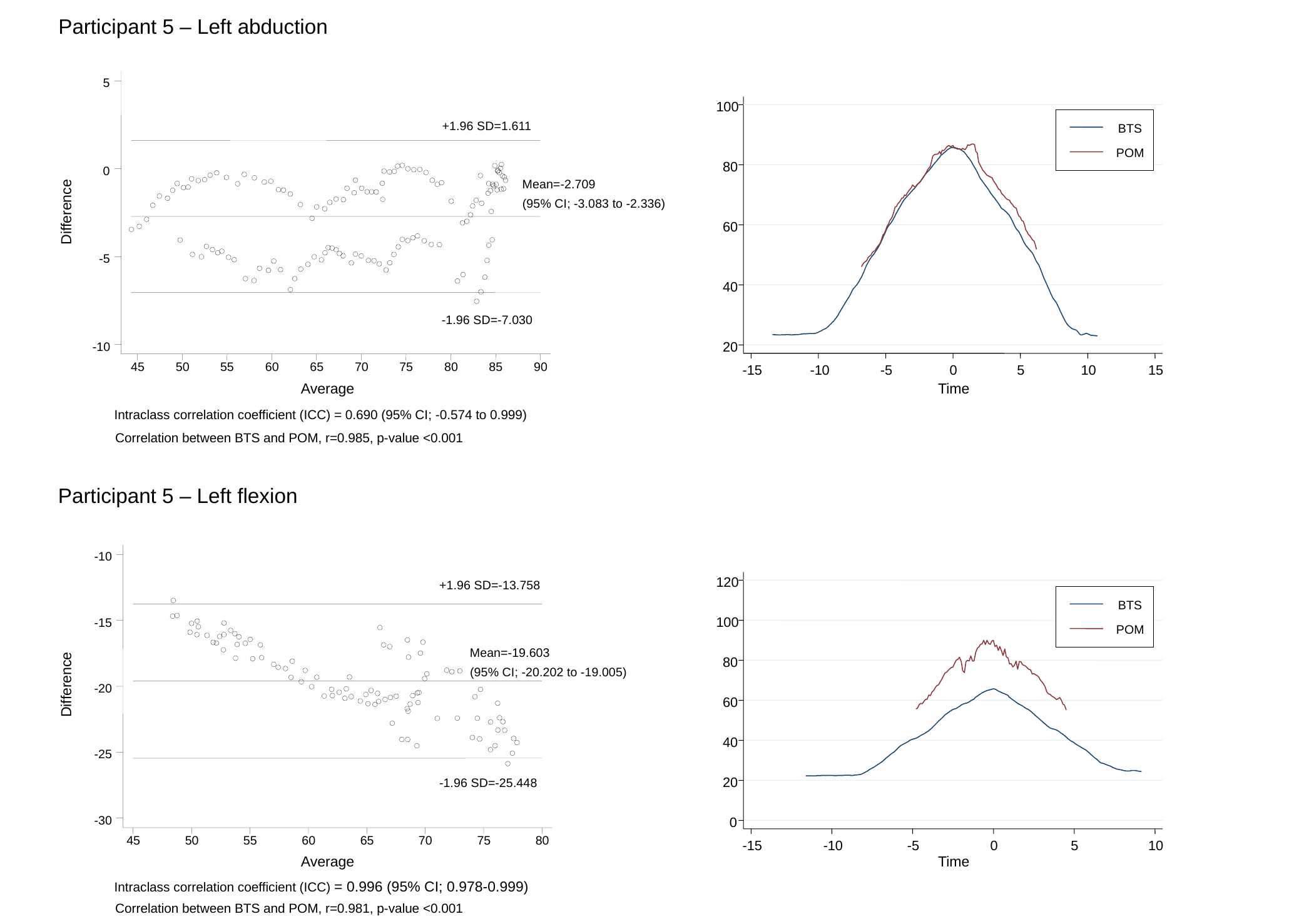

Participant 5 – Left abduction
5
100
BTS
POM
+1.96 SD=1.611
80
0
Mean=-2.709
Difference
Average
Time
(95% CI; -3.083 to -2.336)
60
-5
40
-1.96 SD=-7.030
20
-10
45
50
55
60
65
70
75
80
85
90
-15
-10
-5
0
5
10
15
Intraclass correlation coefficient (ICC) = 0.690 (95% CI; -0.574 to 0.999)
Correlation between BTS and POM, r=0.985, p-value <0.001
Participant 5 – Left flexion
-10
120
+1.96 SD=-13.758
BTS
POM
100
-15
Mean=-19.603
Difference
Average
Time
80
(95% CI; -20.202 to -19.005)
-20
60
40
-25
20
-1.96 SD=-25.448
-30
0
45
50
55
60
65
70
75
80
-15
-10
-5
0
5
10
Intraclass correlation coefficient (ICC) = 0.996 (95% CI; 0.978-0.999)
Correlation between BTS and POM, r=0.981, p-value <0.001

## Slide 11
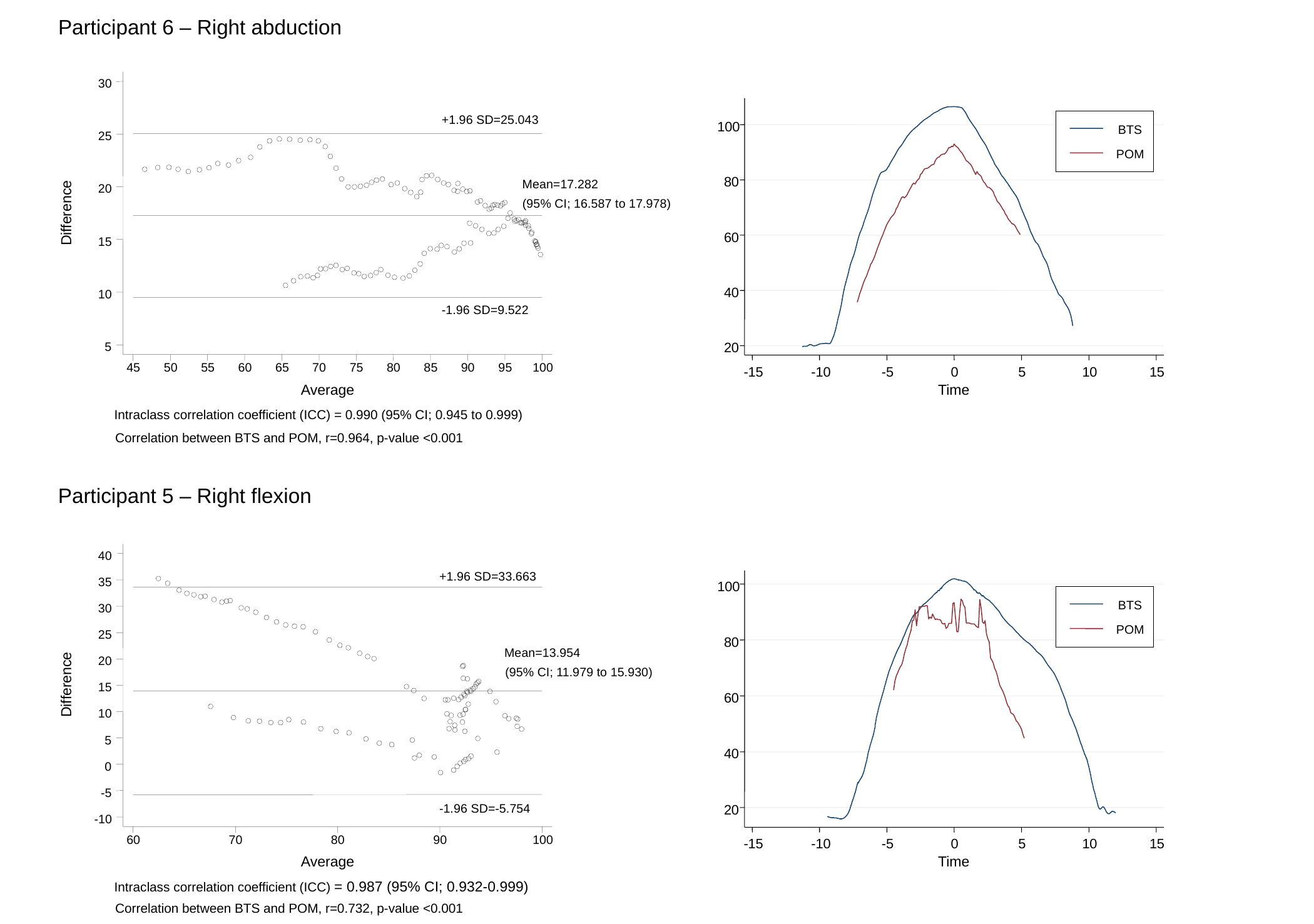

Participant 6 – Right abduction
30
BTS
POM
+1.96 SD=25.043
100
25
80
Mean=17.282
Difference
Average
Time
20
(95% CI; 16.587 to 17.978)
60
15
40
10
-1.96 SD=9.522
5
20
45
50
55
60
65
70
75
80
85
90
95
100
-15
-10
-5
0
5
10
15
Intraclass correlation coefficient (ICC) = 0.990 (95% CI; 0.945 to 0.999)
Correlation between BTS and POM, r=0.964, p-value <0.001
Participant 5 – Right flexion
40
+1.96 SD=33.663
35
100
BTS
POM
30
25
80
Mean=13.954
Difference
Average
Time
20
(95% CI; 11.979 to 15.930)
15
60
10
5
40
0
-5
-1.96 SD=-5.754
20
-10
60
70
80
90
100
-15
-10
-5
0
5
10
15
Intraclass correlation coefficient (ICC) = 0.987 (95% CI; 0.932-0.999)
Correlation between BTS and POM, r=0.732, p-value <0.001

## Slide 12
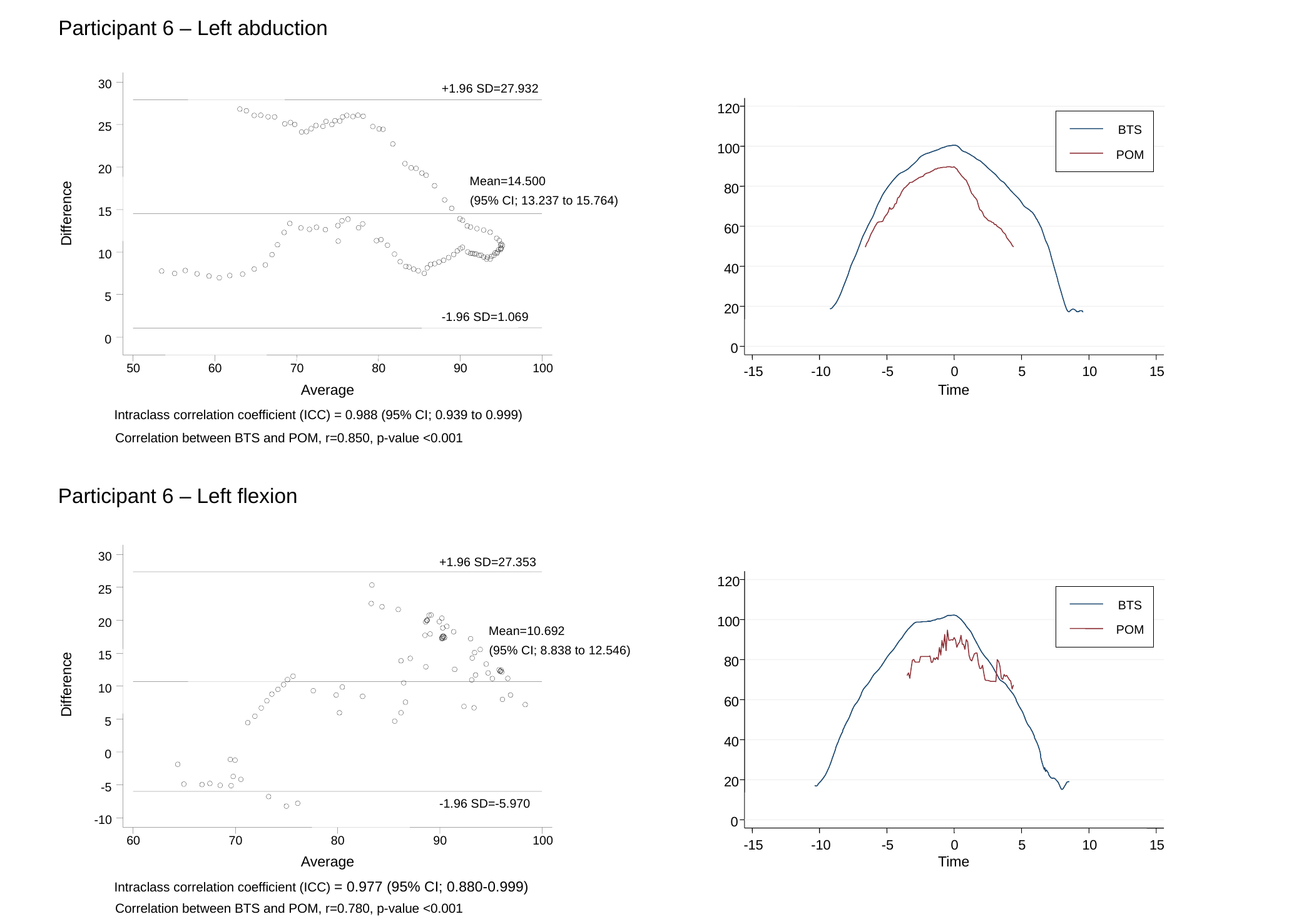

Participant 6 – Left abduction
30
+1.96 SD=27.932
120
BTS
POM
25
100
20
Mean=14.500
80
Difference
Average
Time
(95% CI; 13.237 to 15.764)
15
60
10
40
5
20
-1.96 SD=1.069
0
0
50
60
70
80
90
100
-15
-10
-5
0
5
10
15
Intraclass correlation coefficient (ICC) = 0.988 (95% CI; 0.939 to 0.999)
Correlation between BTS and POM, r=0.850, p-value <0.001
Participant 6 – Left flexion
30
+1.96 SD=27.353
120
25
BTS
POM
100
20
Mean=10.692
(95% CI; 8.838 to 12.546)
15
Difference
Average
Time
80
10
60
5
40
0
20
-5
-1.96 SD=-5.970
-10
0
60
70
80
90
100
-15
-10
-5
0
5
10
15
Intraclass correlation coefficient (ICC) = 0.977 (95% CI; 0.880-0.999)
Correlation between BTS and POM, r=0.780, p-value <0.001
